# PDE4 Inhibition and Inflammatory Bowel Disease: A Novel Therapeutic Avenue

**DOI:** 10.3390/ijms18061276

**Published:** 2017-06-15

**Authors:** Marco Spadaccini, Silvia D’Alessio, Laurent Peyrin-Biroulet, Silvio Danese

**Affiliations:** 1 Laboratory of Gastrointestinal Immunopathology, Humanitas Clinical and Research Center, Milan 20089, Italy; marco.spadaccini@humanitas.it (M.S.); silvia.dalessio@humanitasresearch.it (S.D.); 2 Department of Medical Biotechnologies and Translational Medicine, University of Milan, Milan 20129, Italy; 3 Department of Hepato-Gastroenterology and Inserm U954, University Hospital of Nancy, Lorraine University, Vandoeuvre-lès-Nancy 54500, France; peyrinbiroulet@gmail.com; 4 Department of Biomedical Sciences, Humanitas University, Milan 20089, Italy

**Keywords:** PDE4, Inflammatory Bowel Disease, apremilast

## Abstract

Background. In the last few decades, a better knowledge of the inflammatory pathways involved in the pathogenesis of Inflammatory Bowel Disease (IBD) has promoted biological therapy as an important tool to treat IBD patients. However, in spite of a wider spectrum of biological drugs, a significant proportion of patients is unaffected by or lose their response to these compounds, along with increased risks of infections and malignancies. For these reasons there is an urgent need to look for new pharmacological targets. The novel Phosphodiesterase 4 (PDE4) inhibitors have been recently introduced as new modulators of intracellular signals and gene transcription for the treatment of IBD. Aim. To discuss and describe the state of the art of this new class of compounds in the IBD field, with particular attention to apremilast. Methods. Published articles selected from PubMed were comprehensively reviewed, with key words including apremilast, inflammatory disease, IBD, psoriasis, psoriatic arthritis, pathogenesis, therapies, and treatment. Results. PDE4 inhibitors generate elevated intracellular levels of cyclic Adenosine Monophosphate (cAMP), that consequently down-regulate the release of pro-inflammatory cytokines in the mucosa of IBD patients. The newly developed apremilast is one of these drugs and has already been approved for the treatment of dermatologic/rheumatologic inflammatory conditions; studies in psoriasis and psoriatic arthritis have in fact demonstrated its clinical activity. However, no clinical trials have yet been published on the use of apremilast in IBD. Conclusion. In light of the similarity of pro-inflammatory signaling pathways across the gut, the skin, and joints, apremilast is likely supposed to show its efficacy also in IBD.

## 1. Introduction

Inflammatory Bowel Diseases (IBD) are chronic, progressive, and relapsing disorders involving the human gut. IBD primarly includes Crohn’s disease (CD) and Ulcerative Colitis (UC), that differ in tissue damage distribution and histological features leading to specific clinical symptoms and complications [1,2].

In the last 15 years, a better knowledge of the inflammatory pathways involved in the pathogenesis of these diseases and an inadequate response to conventional treatment have promoted biological therapy as an important tool to treat IBD patients, as shown by anti-Tumor Necrosis Factor (TNF) agents [3,4].

However, in spite of a wider spectrum of biological drugs, a significant proportion of patients are unaffected by or lose their response to these molecules [5,6,7,8,9,10,11], along with increased risks of infections and malignancies [12,13]. For these reasons there is an urgent need to look for new pharmacological targets.

Phosphodiesterase 4 (PDE4) belongs to a group of enzymes that catalyze the breakdown of 3,5′-cAMP (cAMP) in several types of cells, including inflammatory cells, and is considered an important player of the inflammatory cascade. With these premises, drugs targeting these enzymes represent a valuable strategy for the treatment of inflammatory disorders. Among these, apremilast, which specifically target PDE4, has already been approved for the treatment of dermatologic/rheumatologic inflammatory conditions and it is supposed to show efficacy in a wider range of immune-mediated inflammatory diseases.

This review aims to analyze the mechanisms of action of PDE4 inhibitors and to describe the state of the art of apremilast and its similar compounds for the treatment of various inflammatory conditions; we will focus our attention on apremilast and on how its clinical efficacy in patients with Psoriasis and Psoriatic Arthritis (PsA), may be translated in IBD. Furthermore, we report the first attempts in using PDE4 inhibitors for the treatment of intestinal inflammation, both in humans and mice.

## 2. PDE4 and cAMP: Structural and Functional Features

Phosphodiesterase 4 (PDE4) is one of the low *K*_m_ enzymes that catalyzes the breakdown of cAMP in several immune cell types, such as macrophages and T cells [14]; this leads to reduced intracellular levels of cAMP, that is ultimately hydrolyzed to inactive 5′-AMP. In mammalian species there are 11 different families of PDEs (PDE1–11), each containing a conserved catalytic domain, which is structured into three different subdomains for a total of 315 amino acids. The four gene families (A, B, C, and D) encoding for PDE4 can produce multiple protein products by alternative mRNA splicing, thus generating approximately 20 different PDE4 variants, each containing a unique N-terminal region [15,16,17], important for the subcellular localization and the functionality of single isoforms [18].

A better understanding of how PDE4 isoforms interact with other proteins and of the mechanisms governing the catalytic activity of these enzymes has been reached through the X-ray crystal determination of the structure of an active PDE4B catalytic unit; however detailed molecular/structural information is not yet available for the protein in its entirety [18].

Next to the catalytic domain there are additional Upstream Conserved Regions, UCR1 and UCR2, that appear to be fundamental for the regulation of PDE4 functionality. While UCR1 contains the Protein kinase A (PKA) phosphorylation site, which is important for the regulation of PDE4 catalytic function, UCR2 is characterized by an auto-inhibitory capability, that is responsible for the modulation of PDE4 enzymatic activity [19,20].

Moreover, on the basis of the presence and the characteristics of these highly conserved regions, the PDE4 subfamily can be divided into three forms: the “super-short” form, which contain only the C-terminal portion of UCR2; the “short” form without UCR1, and the “long” form which contain both UCR1 and UCR2 [21,22]. UCR1 and UCR2 in PDE4 long forms can interact with each other leading to PDE4 dimerization, whereas the short forms are presented as monomers. Disruption of dimerization has been shown to decrease the (PKA) phosphorylation-dependent activation of PDE4 and to reduce the affinity to rolipram, one of the first generated PDE4 inhibitors [23,24].

Cyclic Adenosine Monophosphate (cAMP) and Guanosine Monophosphate (cGMP) are two cyclic nucleotides able to work as secondary messengers, that modulate gene expression by naturally translating extracellular stimuli into intracellular signaling [25]. cAMP is generated at the cytosol surface of the plasma membrane by the adenylate cyclase, an enzyme of pivotal importance in determining several aspects of cellular function, including heart rate and contractility, smooth muscle relaxation, and immune responses [26,27,28,29]. Ligands such as leukotrienes, prostaglandins, chemokines, and histamine are able to bind to G-protein coupled receptors and to activate adenylate cyclase, which consequently increases cAMP production [30] and its interaction with proteins such as PKA.

There is a lot of evidence about the different distributions of adenylate cyclase and receptors able to stimulate this enzyme [18,31]; moreover, different pools of PKA or PDEs localize in distinct intracellular sites in order to tailor cAMP production, function, and degradation [28,32]. Therefore, it is not unusual to find PDE activity in a variety of membranes and other subcellular locations, other than the cytosol, and it is now recognized that cAMP-activated signaling is compartmentalized [17,18,28,32]. The wide distribution of the PDE4 isoenzyme in mammalian cells, justify the tissue-specific biological functions of this protein [18].

## 3. From the Pathogenetic Pathway to a New Therapeutic Target

Considering that the Nuclear Factor κ-light-chain-enhancer of activated B cells (NF-κB) has the capability to mediate specific effects of different cell-types, pharmacological attempts to block its activation represents new therapeutic strategies in IBD [28,33,34].

PDE4 inhibitors are considered potential suppressors of NF-κB-dependent inflammatory conditions. In fact, for decades cAMP has been known for its role in interfering with NF-κB signaling, thus being recognized as an anti-inflammatory and immunosuppressive player [35]. Consequently, PDE4 that controls the concentration of cAMP, might indirectly influence the activation of NF-κB signaling pathway under inflammatory conditions.

For example, in both monocytes and endothelial cells, high levels of cAMP sustained by forskolin, an adenylate cyclase activator, have the power to directly inhibit NF-κB-dependent gene transcription [36], without any effect on nuclear translocation nor phosphorylation of the NF-κB subunit p65 [36]. In macrophages chronically treated with ethanol, and upon Lipopolysaccharide (LPS) stimulation, PDE4 inhibition has been shown to reduce the TNF-α mRNA expression, again by a mechanism that involves transcriptional modulation of NF-κB [37]. This mechanism was confirmed on LPS-stimulated murine macrophages by roflumilast, a selective PDE4 inhibitor able not only to promote in these cells high levels of intracellular cAMP, but also to inhibit the production of nitric oxide, and the expression of TNF-α and inducible nitric oxide synthase (iNOS), both in a NF-κB-dependent manner [38,39].

Inhibition of PDE4 leads not only to reduced NF-κB-mediated TNF-α expression, but also to increased synthesis of IL-10, an anti-inflammatory cytokine, through PKA activation [40]. This dual effect is due to the presence of multiple cAMP responsive elements (CREs) within the *IL-10* promoter, that may recruit different substrates of PKA, such as the two CRE binding proteins, cAMP Response Element-Binding protein (CREB) and cyclic AMP-dependent Transcription Factor-1 (ATF-1) [41].

Thus, PDE4 inhibitors may act both negatively or positively on gene expression, on the basis of different CRE elements located within the gene promoter.

Modulation of the NF-κB pathway by PDE4 and cAMP has also been observed in T cells, where PDE4 seems to control not only the release of TNF-α, IL-2, IL-4, and IL-5, but also the expansion of T lymphocytes per se, as demonstrated by the capacity of rolipram to suppress antigen-induced proliferation of T cells [42,43]. Moreover, while high levels of PDE4 were found to promote T-cell receptor/CD28-stimulated cytokine production, the inhibition of PDE4 in T lymphocytes blocked NF-κB signaling [44].

TH1 and TH2 clonal cell proliferation and cytokines production are both inhibited by rolipram, with the TH2 subpopulation being more sensitive to PDE4 inhibition [45,46].

cAMP has also been described as a key mediator of naturally occurring regulatory T cell (nTreg) suppression, by crossing the cell membrane of responder T cells, such as CD4-positive [47] and TH2 subsets [48], and inhibiting T cell proliferation. This is important because the use of PDE4 inhibitors in auto-immune or immune-mediated inflammatory disorders may increase cAMP levels into responder T cells and block their expansion. Bopp and colleagues indeed demonstrated that rolipram was able to significantly block TH2 responses both in vitro, by co-culture with nTreg, and in vivo using two different mouse models of TH2-dependent airway inflammation and hyperresponsiveness [48], thus highlighting the relevance of using PDE4 inhibitors in human inflammatory diseases.

The TH17 subset of T lymphocytes, thought to be crucial for the pathogenesis of autoimmune diseases, produce IL-17A and IL-17F, which amplify the inflammatory cascade directly or indirectly acting on neutrophils and causing their recruitment to the inflammation site. On these cells PDE4 inhibitors profoundly attenuate IL-17 production [49,50].

In neutrophils and eosinophils, PDE4 promotes both chemotaxis and degranulation. In neutrophils this is mediated by the ability of PDE4 to stimulate the release of leukotriene B4, IL-8, and superoxide anions. Furthermore, PDE4 has been found to control neutrophil adhesion to vascular endothelial cells, by inducing the expression of important adhesion molecules, such as the β2-integrin Mac-1 [21,51,52].

In non-immune cells, such as the blood endothelium, PDE4 inhibitors exert anti-angiogenic effects; this was observed in terms of reduced E-selectin expression on endothelial cells [53], as well as decreased Vascular Endothelial Growth Factor (VEGF)—induced endothelial cell migration [54,55].

Overall, these studies underline the mechanisms through which PDE4 inhibitors might exert their anti-inflammatory effects, acting on non-immune cells but particularly on both the innate and adaptive immune system, suppressing the release of pro-inflammatory cytokines and stimulating the secretion of anti-inflammatory molecules, thus shifting the immune balance toward an anti-inflammatory status.

## 4. PDE4 Inhibitors in Preclinical Studies of IBD

Animal studies have shown beneficial effects of PDE4 inhibitors, such as rolipram, mesopram, roflumilast, and tetomilast in experimental models of colitis. For example, roflumilast has been shown to ameliorate not only the clinical score, but also the expression of TNF-α in murine colitis [56]. Clinical signs of intestinal inflammation were also significantly reduced by rolipram in both mice and pigs, in comparison with untreated animals [57,58]. Moreover, in these models rolipram suppressed the release of colonic TNF-α, improved the histological score, and reduced collagen production, being effective in both the prevention of intestinal fibrosis and the treatment of established colitis [57,58,59,60,61]. In addition, this drug increases the release of IL-10, and inhibits the proliferation of primary T cells mediated by IL-2 [42].

Mesopram has been shown to ameliorate dextran sulfate sodium (DSS)-induced murine colitis, in terms of reduced clinical and histologic scores and decreased colonic INF-γ; this was confirmed both in preventive and therapeutic settings [62].

Another PDE4 specific inhibitor, tetomilast, was initially identified for its properties in stimulating the production of anti-inflammatory molecules in neutrophils, through in vitro screening of compounds derived from thiazole [63]. In preclinical studies it has been shown to inhibit the release of proteases, superoxide anions, and other functions attributable to activated human leukocytes [63]. Although its mode of action has not been completely understood, when tested on mouse or rat models of colitis and, in vitro, on human immune cells, it suppressed LPS-induced production of TNF-α and IL-12 in monocytes, and both TNF-α and INF-γ in CD4-positive T cells, leading to reduced colonic damages [64].

Furthermore, in animal models of IBD, tetomilast was able to prevent gut barrier damage or the loss of epithelial structure/function; this was due to its action on F-actin depolymerization, which in these models occurs under a low level of cAMP [64].

Apremilast, also known as CC-10004 (CAS registry number 608141-41-9), is one of the most recent PDE4 inhibitor developed, able to block cAMP degradation by binding to the catalytic site of PDE4 enzyme. This drug was found to significantly enhance anti-inflammatory CREB-dependent genes transcription in Jurkat T cells and THP-1 monocytes, promoting a higher transcription of *IL-10* and *IL-6* [35]. In T cell cultures, the drug also inhibits IL-2 production, but influences also the release of IL-5 and IL-13 by TH2, IL-17 by TH17, and TNF-α, Granulocyte-Macrophage Colony-Stimulating Factor (GM-CSF), and interferon-γ by TH1 subsets. On the contrary, the clonal expansion of T- or B-cells seems not to be affected by apremilast, as well as immunoglobuline production [35].

Upon LPS challenge, apremilast has been shown to block the release of TNF-α, IFN-γ, and IL-12p70, as well as the chemokines (C-X-C motif) ligand 9 (CXCL9), CXCL10, and (C-C motif) Ligand 4 (CCL4), from human peripheral blood mononuclear cells [65]. It also inhibits the adhesion of polymorphonuclear cells to human microvascular endothelium by down-regulating the expression of CD18 and CD11b and the recruitment of neutrophils towards inflamed tissues, by reducing the production of molecules that are considered chemoattractant for these cells, such as IL-8 or Leukotriene B4 [66].

**Figure 1:**
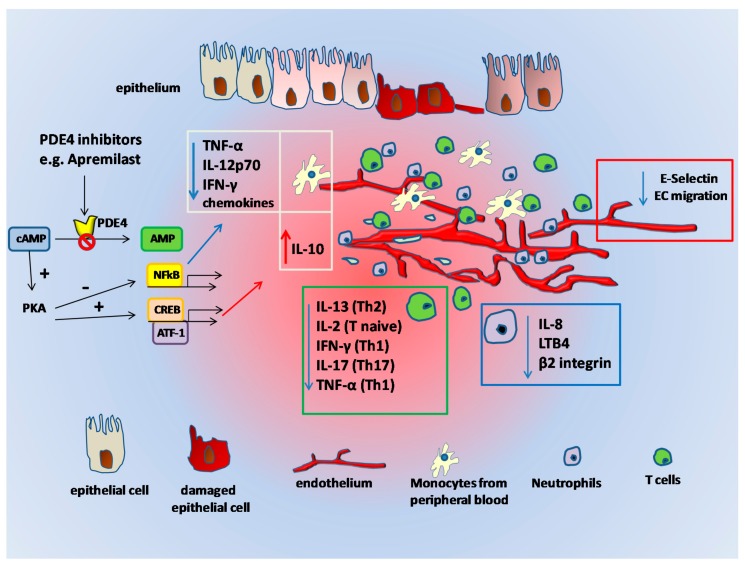
Schematic representation of how Phosphodiesterase 4 (PDE4) inhibitors, e.g., apremilast, exert their functions within the inflamed mucosa. In several cell types populating the inflamed gut, such as monocytes, T cells, neutrophils, and endothelial cells, cyclic Adenosine Monophosphate (cAMP) is degraded to AMP mainly by PDE4. PDE4 inhibition by apremilast and similar compounds increases intracellular cAMP levels and determines the activation of Protein Kinase A (PKA). PKA activation induces the phosphorylation of transcription factors such as CREB, that in turn binds the promoters of genes encoding from various anti-inflammatory (IL-10) cytokines. Similarly, the presence of other coactivators may influence PKA activity, resulting in the inhibition of Nuclear Factor κ-light-chain-enhancer of activated B cells (NF-κB) transcriptional activity and reduced expression of specific pro-inflammatory cytokines and chemokines. PDE4 inhibitors have also been shown to reduce the expression of E-selectin on endothelial cells, thus reducing angiogenesis. Blue and red arrows: downregulation and upregulation of the indicated molecules, respectively.

In lamina propria mononuclear cells derived from the intestinal mucosa, apremilast reduces the release of TNF-α. Furthermore, in these same cells isolated from CD or UC patients, it restrains the release of Matrix Metalloproteinase-3 (MMP-3), one of the metalloproteinases that controls the TNF-α-induced mucosal damage in IBD [67]. This intricate network derived from the use of PDE4 inhibitors, such as apremilast (Figure 1), illustrates the capability of this drug to interfere with different points of the cascade that causes gut tissue damage, and emphasizes the prospect of using apremilast to treat inflammatory disorders of the gut.

## 5. Apremilast in Immune-Mediated Inflammatory Disorders

In the last two decades many PDE4 selective inhibitors have been studied, and some of them have been evaluated in clinical trials for several inflammatory conditions, such as IBD, asthma, Chronic Obstructive Pulmonary Disease (COPD), atopic dermatitis, multiple sclerosis, psoriasis, Psoriatic Arthritis (PsA), and rheumatoid arthritis [14]. However, the development of most of these drugs has been discontinued because of narrow therapeutic windows, mostly depending on dosing limitations caused by side effects such as nausea and emesis.

In psoriasis-affected patients for example, PDE4 has been shown to be expressed in all inflammatory cells involved in the pathophysiologic processes of this disease, including the production of cytokines such as TNF-α, IL-12, and IL-23 by antigen-presenting cells, and the synthesis of IL-2, IL-5, and IFN-γ by T cells [68]. Moreover, PDE4 seems to influence also NF-κB expression, TNF-α production, and regulation of IL-8 by keratinocytes [69].

In a pre-clinical in vivo model of psoriasis, performed using human skin xeno-transplanted onto Severe Combined Immunodeficient (SCID) mice, followed by the administration of human natural killer cells, apremilast significantly reduced keratinocyte growth, skin thickness, and histopathologic features of the disease, along with a diminished expression of TNF-α, Intercellular Adhesion Molecule-1 (ICAM-1), and Human Leukocyte Antigen-D Related (HLA-DR) in the skin grafts [66].

In the last few years, thanks to its action in reducing epidermal thickness, Keratin 16 expression, and lymphocyte infiltration [70], the use of oral apremilast (Otezla^®^) was approved by the United States Food and Drug Administration (FDA) for the treatment of moderate-to severe plaque psoriasis in September 2014, as well as by the European Commission in January 2015. Patients treated with this drug display significant changes in plasma levels of pro-inflammatory TNF-α, IL-8, IL-17, and IL-23, with a reduced number of leukocytes infiltrating the psoriatic lesions [71,72]. Five additional randomized controlled trials have been published about the safety and efficacy of apremilast in psoriasis [73,74,75,76,77] (Table 1). In the two phase III clinical trials called “Efficacy and Safety Trial Evaluating the Effects of apremilast in Psoriasis” (ESTEEM), a 75% (ESTEEM 1) or a 50% (ESTEEM 2) reduction from baseline in the PASI score (i.e., a PASI-75 or -50 response) has been achieved significantly more by apremilast than placebo recipients at Week 16 [74,76]. Furthermore, the beneficial effects exerted by apremilast on these psoriatic patients were independent from their baseline features, such as disease duration, PsA history, prior psoriasis therapies, or response to such therapies. Additional data from these trials revealed that apremilast has long term benefits on the skin and other psoriatic manifestations of these patients [74,76,78].

Similarly, in another recent phase IIIb, randomized, double-blind, placebo-controlled study (LIBERATE), apremilast demonstrated significant efficacy vs. placebo at Week 16 in biologic-naive patients with psoriasis, which was sustained over 52 weeks, thus being consistent with the known safety profile of apremilast in these patients [79].

Psoriatic arthritis (PsA) also shares with psoriasis and IBD several pathogenic mechanisms that engage both the innate and adaptive immune cell compartments, with a particular role exerted by increased T cell activation and recruitment [80]. In fact, in PsA, lymphocytic infiltrate invades joints and releases high levels of TNF-α, IL-1β, interferon-γ, and IL-2, which in turn sustain synovial growth and joint damage [80].

The use of oral apremilast (Otezla^®^) was approved by the FDA in March 2014, as well as by the European Commission in January 2015, for the treatment of adults with active PsA. Its clinical efficacy was observed in both phase II [77,81] and phase III trials (the Psoriatic Arthritis Long-term Assessment of Clinical Efficacy: PALACE 1, 2, and 3) [82,83,84], in which 41% of patients achieved ACR20 (20% improvement in American College of Rheumatology criteria) for apremilast 30 mg twice daily, compared with 18% for the placebo group [81], with clinical improvements generally being sustained for up to Week 104 [82]. Apremilast has also demonstrated beneficial effects in disease-modifying anti-rheumatic drugs (DMARDs)-naïve PsA-affected patients in the phase III trial, named PALACE 4 [85], although its use is not currently approved in this setting in the EU (Table 1).

In general, psoriasis- and PsA-affected patients enrolled in Phase III clinical studies well tolerated apremilast; however, disorders of the gastrointestinal tract, such as diarrhea (15.7%) and nausea (13.9%), have been observed within the first 2 weeks of treatment, with mild to moderate signs of severity. These side effects were reported to be severe only in 0.3% of patients. Other commonly described adverse reactions included infections of the upper respiratory tract (8.4%), headache (7.9%), and tension headache (7.2%). Even though data are currently limited, apremilast has rarely been associated with laboratory abnormalities and does not seem to elevate the risk of malignancies, severe infections, or other cardiac adverse effects [86].

Although preclinical studies have shown therapeutic effects of PDE4 inhibitors, such as rolipram, mesopram, and tetomilast in experimental colitis, until now human studies have failed to show any success in the treatment of IBD [65,87], and nothing have been published on the use of apremilast.

In the first Phase II randomized placebo controlled double-blind study on 186 patients with mild to moderate UC [87], tetomilast-treated patients showed no significant improvement in disease activity index, when compared to the placebo. The active group showed a high drop-off rate, mostly due to self-limiting upper gastrointestinal symptoms such as nausea and vomiting that decimated the subjects available for the data analysis. However, a post hoc analysis on secondary endpoints showed that UC patients with high disease activity scores respond better to tetomilast, suggesting potential clinical efficacy [87].

The Phase III study did not show any significant difference between tetomilast and placebo at both primary and secondary end points, but revealed a trend toward decreasing the severity of bleeding in the tetomilast group [65].

Overall, these findings suggest that although PDE4 inhibitors can be considered as multicytokine blockers with potential therapeutic effects in IBD, further studies are required to improve this type of approach to medical treatment in IBD patients. Pharmaceutical research in the field of chronic inflammatory disorders has made a step forward by developing new PDE4 inhibitors with a higher therapeutic index [88]. Apremilast, introduced to the clinic [35,89] and approved in the USA for the treatment of PsA [90], is one of these novel drugs, thus representing a promising tool for the medical treatment of other diseases mediated by the immune system.

## 6. Conclusions

On the whole, preclinical and clinical findings reported in this review highlight a remarkable role for PDE4 in the events responsible for the onset of chronic inflammation in IBD. Elevated intracellular cAMP levels generated by PDE4 inhibitors lead to downregulation of the inflammatory response by modulating the expression of NF-κB, TNF-α, IL-1β, IL-17, and other pro-inflammatory molecules found to be released in the mucosa of patients with IBD. These mechanisms highlight the potential of PDE4 inhibitors in the treatment of IBD. Of note, although several clinical and molecular aspects distinguish CD from UC patients, these disorders shared many pro-inflammatory molecules; this aspect increases the probability that a small-molecule inhibitor of PDE4, such as apremilast, may be effective for the treatment of both forms of IBD.

Increasing evidence for the clinical efficacy and safety outcomes of apremilast have been reported for the treatment of inflammatory disorders in other organs. In light of the similarity of pro-inflammatory signaling pathways across the gut, the skin, and joints, translation of therapeutic approaches from other immune-mediated inflammatory disorders, such as Psoriasis and PsA, to the intestine appears to be a promising treatment strategy. Moreover, apremilast has the advantage of not showing any selectivity for other PDE4 subfamily members (e.g., PDE4 A4, B2, C2, and D3), which may in part justify its greater therapeutic index in comparison with other PDE4 inhibitors in non-clinical models [69].

Given the fact that a high percentage of IBD patients are unaffected by or lose their response to the current therapeutic armamentarium, there remains an unmet need for novel treatment options. Apremilast, with its multi-cytokine blocking activity, represents an attractive candidate which could lead to a more rational treatment of patients with IBD in the future.

## 7. Methods

A PubMed search was performed focusing mostly on full-text papers written in the English language; abstracts were considered when the full-text was not available. The database was searched up to October 2016 using the following words, individually or in combination: “apremilast”, “tetomilast”, “inflammatory disease”, “IBD”, “ulcerative colitis”, “psoriasis”, “psoriatic arthritis”, “pathogenesis”, “inflammation pathway”, “therapies”, and “treatment”. Publication data restrictions were not applied.

## Figures and Tables

**Table 1:** Summary of clinical trials with apremilast for Psoriasis and Psoriatic Arthritis.

Disease	Trial Number	Patients	Design	Outcome	Reference
**Psoriasis**	NCT00604682	19	Phase II, Open-label, 29 days	Improved PASI, Reduced epidermal thickness and T cells	Gottlieb at al. [70]
	NCT00521339	30	Phase II, Open-label, 12 weeks	Improved PASI, Reduced myeloid DC, T- and NK-cells	Gottlieb at al. [72]
	NCT00773734	89	Phase IIb, Randomized, DB, PC, 16 weeks; Open-label, additional 8 weeks	Improved PASI at 20 and 30 mg	Papp et al. [73]
	NCT00606450	259	Phase II, Randomized, DB, PC, 12 weeks	Improved PASI and reduced mean body surface area involvement	Papp et al. [75]
	NCT00773734	352	Phase IIb, Randomized, DB, PC, 16 weeks	Improved DLQI score and pruritus	Strand et al. [77]
	NCT01194219 (ESTEEM 1)	844	Phase III, Randomized, DB, PC, 16 weeks	Improved PASI	Papp et al. [74]
	NCT01232283 (ESTEEM 2)	413	Phase III, Randomized, DB, PC, 16 weeks	Improved PASI	Paul et al. [76]
	NCT01690299 (LIBERATE)	250	Phase IIIb, Randomized, DB, PC, 52 weeks	Improved PASI	Reich et al. [79]
**Psoriatic Arthritis**	NCT00456092	204	Phase II, Randomized, DB, PC, 12 weeks	Improved ACR20 at 20 and 40 mg	Schett et al. [81]
	NCT01172938, NCT01212757, NCT01212770, NCT01307423 (PALACE 1-4)	2026	Phase III, Randomized, DB, PC, 16 weeks	Improved ACR20, symptoms and PASI scores	Poole and Ballantyne [82,83,84,85]

ACR20, American College of Rheumatology criteria for 20% improvement; DB, double-blind; DC, dendritic cells; NK, Natural Killer; DLQI, Dermatology Quality of Life Index; PASI, Psoriasis Area and Severity Index; and PC, placebo-controlled.

## Abbreviations

ACR20	American College of Rheumatology Criteria for 20% Improvement
DB	Double-Blind
DC	Dendritic Cells
NK	Natural Killer
DLQI	Dermatology Quality of Life Index
PASI	Psoriasis Area and Severity Index
PC	Placebo-Controlled
